# Efficient CdTe Nanocrystal/TiO_2_ Hetero-Junction Solar Cells with Open Circuit Voltage Breaking 0.8 V by Incorporating A Thin Layer of CdS Nanocrystal

**DOI:** 10.3390/nano8080614

**Published:** 2018-08-13

**Authors:** Xianglin Mei, Bin Wu, Xiuzhen Guo, Xiaolin Liu, Zhitao Rong, Songwei Liu, Yanru Chen, Donghuan Qin, Wei Xu, Lintao Hou, Bingchang Chen

**Affiliations:** 1 School of Materials Science and Engineering, South China University of Technology, Guangzhou 510640, China; mxianglin@sina.com (X.M.); damien_wu@foxmail.com (B.W.); XzGuo_19@163.com (X.G.); LiuLin9708@163.com (X.L.); rzt1512388848@163.com (Z.R.); majestyv@sina.com (S.L.); caro_ccyr@163.com (Y.C.); 17728100969@163.com (B.C.); 2 Institute of Polymer Optoelectronic Materials & Devices, State Key Laboratory of Luminescent Materials & Devices, South China University of Technology, Guangzhou 510640, China; 3 Guangdong Provincial Key Laboratory of Optical Fiber Sensing and Communications, Guangzhou Key Laboratory of Vacuum Coating Technologies and New Energy Materials, Siyuan Laboratory, Department of Physics, Jinan University, Guangzhou 510632, China

**Keywords:** nanocrystal, CdTe, TiO_2_, CdS, solar cells, solution processed

## Abstract

Nanocrystal solar cells (NCs) allow for large scale solution processing under ambient conditions, permitting a promising approach for low-cost photovoltaic products. Although an up to 10% power conversion efficiency (PCE) has been realized with the development of device fabrication technologies, the open circuit voltage (*V_oc_*) of CdTe NC solar cells has stagnated below 0.7 V, which is significantly lower than most CdTe thin film solar cells fabricated by vacuum technology (around 0.8 V~0.9 V). To further improve the NC solar cells’ performance, an enhancement in the *V_oc_* towards 0.8–1.0 V is urgently required. Given the unique processing technologies and physical properties in CdTe NC, the design of an optimized band alignment and improved junction quality are important issues to obtain efficient solar cells coupled with high *V_oc_*. In this work, an efficient method was developed to improve the performance and *V_oc_* of solution-processed CdTe nanocrystal/TiO_2_ hetero-junction solar cells. A thin layer of solution-processed CdS NC film (~5 nm) as introduced into CdTe NC/TiO_2_ to construct hetero-junction solar cells with an optimized band alignment and *p*-*n* junction quality, which resulted in a low dark current density and reduced carrier recombination. As a result, devices with improved performance (5.16% compared to 2.63% for the control device) and a *V_oc_* as high as 0.83 V were obtained; this *V_oc_* value is a record for a solution-processed CdTe NC solar cell.

## 1. Introduction

Since the first reported solution-processed CdTe nanocrystal solar cells (NCs) in 2005, they have been rapidly developed due to their potential for next-generation photovoltaic products (including NCs, quantum dots, polymers, Sb_2_Se_3_, and perovskite solar cells) at low cost, low material consumption, and simple fabricating techniques [1,2,3,4,5,6,7,8]. During the past decade, intensive research has been focused on preparing high-quality CdTe NC films to improve the performance of NC solar cells [9,10,11]. Advances in CdTe NC thin film treatment and device architecture have led to a significant increase in the performance of solar cells from 2.9% in 2005 to ~7% in 2011 [12]. Efficient CdTe NC solar cells are prepared by using a planar *p*-*n* hetero-junction configuration. In this device structure, carriers are mainly generated in the CdTe NC film and electrons are injected from the conducting band of the CdTe NC to an n type partner (such as CdSe, CdS, ZnO, or TiO_2_ et al.), while the hole travels to the back contact of the device. Nowadays, solution-processed CdTe NC solar cells mainly suffer from a low open circuit voltage (*V_oc_*): most CdTe NC solar cells have a *V_oc_* between 0.5 V and 0.7 V [13], while these values are 0.8 V~0.9 V for CdTe thin film solar cells that have been prepared by the close space sublimation (CSS) method [14], which limits further improvement in performance. The loss in potential for CdTe NC solar cells is defined as *E_loss_* = *E_g_* − e*V_oc_*, where *E_g_* is the bandgap of the CdTe NC thin film (~1.45 eV). The value of *E_loss_* is greater than 0.7 eV for the CdTe NC solar cells, while this value is below 0.4 eV for most perovskite or III–V group semiconductor solar cells [15,16]. According to the Shockley–Queisser constraint, the minimum *E_loss_* is about 0.3 eV for CdTe NC solar cells and the maximum theoretical *V_oc_* with a bandgap of 1.45 eV is 1.15 eV [17]. The *E_loss_* for CdTe NC solar cells can be mainly attributed to the recombination existing in the *p*-*n* junction and the back contact, given that the CdTe NC film has been prepared at optimized conditions. To obtain low resistance ohmic contacts to CdTe thin films, a heavily doped region at the surface of the CdTe should be formed before back contact formation via wet etching (using a bromine/methanol or phosphoric/nitric treatment) [18,19,20]. Unfortunately, the wet etching will result in the NC thin film being removed from the substrate or device shunt, which was confirmed by Panthani et al. [21].

Another way to make good ohmic contacts to the CdTe NC film is by using metal/p^+^ semiconductor/metal oxide/organic hole transport materials with a high work function as a back contact. Occasionally, Au is selected as the back contact for CdTe NC solar cells, and a *V_oc_* of 0.65 V can be obtained [22] due to the low work function (5.1 eV for Au~5.5 eV for CdTe). Recently, Kurley et al. demonstrated ohmic contacts could be realized by inserting transparent ZnTe:Cu, etched CdTe:Cu, or a Te buffer layer between the CdTe and ITO (Indium Tin Oxide) [23]. Unfortunately, although as high as 8.6% (without light soaking/current treatment) of the PCE coupled with a high fill factor (~60%) and *J_sc_* were attained in this case, the *V_oc_* was below 0.7 V, which limits the device’s performance for further improvement. *p*-doping spiro-OMeTAD [24] or P3KT [25] have also been employed as hole transport materials for the CdTe NC thin film’s back contact, and a high efficiency (~6%) was obtained in optimized NC solar cells. Most recently, a novel crosslinkable conjugated polymer poly(diphenylsilane-co-4-vinyl-triphenylamine) (Si-TPA) with high work function (5.38 eV) was introduced successfully into solution-processed CdTe/CdSe (or CdS) NC solar cells with an inverted structure of (ITO/ZnO/CdSe/CdTe/Si-TPA/Au); a PCE as high as 8.34% was obtained due to the decreased carrier recombination and dipole effects [26]. Another important issue for increasing the *V_oc_* of CdTe NC solar cells is preparing a high-quality *p*-*n* junction and optimizing the band alignment of the whole device. As the size of a CdTe NC is in the range of 1~0 nm, the *n*-type partner is expected to have a similar size to obtain a homogeneous interface. In our previous work, we found that using solution-processed CdS NC or CdSe NC to replace the widely used CBD–CdS (chemical bath deposition CdS) as an *n*-type partner for CdTe NC solar cells improved *V_oc_* and performance due to the high junction quality and reduced carrier recombination in the *p*/*n* junction [27,28]. Most recently, we found that a higher *V_oc_* (0.66–0.74 V) could be obtained in CdTe NC/TiO_2_ heterojunction solar cells by using Sb doped TiO_2_ as the buffer layer due to the improved band alignment. However, the large differences in crystal type (solution-processed TiO_2_ has an anatase structure [29] while CdTe NC has a zinc blende structure [30]) and lattice constant (0.948 nm for TiO_2_ and 0.648 nm for CdTe) resulted in low junction quality. On the contrary, when compared to TiO_2_, CdS had a lower lattice mismatch with CdTe and a high-quality hetero-junction is expected by incorporating a CdS thin film, which suppresses the leakage current due to the reduced defect density. In this paper, we developed an efficient method to simultaneously enhance the *V_oc_* and PCE of solution-processed CdTe NC/TiO_2_ solar cells by inserting a thin layer of CdS NC between the CdTe and TiO_2_ film. The CdS NC possesses a similar size and structure as that of CdTe NC, which can efficiently decrease the lattice mismatch between CdTe and TiO_2_; in addition, CdS has suitable energy levels, which are well matched with CdTe, therefore decreasing the energy loss and improving the *V_oc_* of the device. The incorporation of a CdS NC thin film optimizes the band alignment of the CdTe/TiO_2_ junction and reduces the interface recombination. Compared to the control device (with the structure FTO(SnO_2_:F)/TiO_2_/CdTe/Au), all of the devices with a CdS interlayer showed a significantly higher *V_oc_* (0.72–0.83 V). A *V_oc_* as high as 0.83 V was obtained with an optimal thickness of the CdS NC film (3.74 nm), which is a record for a solution-processed CdTe NC solar cell. When further optimizing the device fabrication conditions, we achieved CdTe/TiO_2_ NC solar cells that exhibited a *J_sc_* of 17.38 mA/cm^2^, a *V_oc_* of 0.73 V, an FF (fill factor) of 40.67%, and a high PCE of 5.16%. This PCE value was almost two times higher than the control device (with a PCE of 2.65%). As a simple fabrication process, we believe that this design holds potential for efficient CdTe NC solar cells with a PCE of up to 10%.

## 2. Experiment Procedure

A TiO_2_ sol-gel precursor was synthesized via a convenient method according to our previous work [31]. In a typical process, 4.25 mL titanium *n*-butoxide, 3.75 mL ethanolamine, and 25 mL ethyl alcohol were mixed and gently stirred in a 50 mL beaker for 2 h to form a transparent sol-gel. Next, 5 mL of acetic acid in 5 mL of deionized water was gradually dropped into the mixture and continuously stirred for 24 h. Finally, the mixture was transferred to the fume hood to accelerate the condensation procedure. When the total volume of the mixture decreased to 15 mL, it was taken out for the fabrication of TiO_2_ thin film. The synthesis of the CdS NC and CdTe NC solutions was conducted following methods published previously [23,28]. Transmission electron microscope (TEM) images of the CdS and CdTe NC are presented in Figure S1a,b. The CdS NC showed a spherical morphology while the CdTe NC showed a rod-shaped structure. The transmission spectrum of FTO/TiO_2_/CdS with different thicknesses of CdS is shown in Figure S2. It is evident that the introduction of a thin layer CdS NC film had little impact on the transmission of FTO/ TiO_2_ (less than 10% decrease when compared to the NC device without CdS NC film), which is prospective for increasing the spectrum response in short wavelengths.

**Figure 1:**
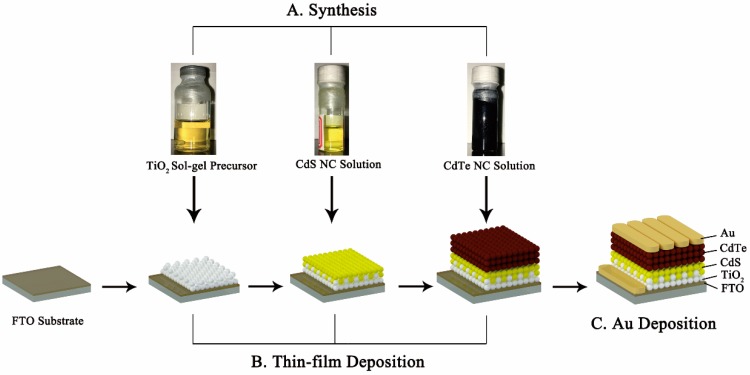
A schematic of the fabrication process of the NC solar cells.

Solar cells with the configuration of FTO/TiO_2_/CdS/CdTe/Au were prepared by a simple solution process under ambient conditions, as shown in Figure 1. A TiO_2_ film with a thickness of 40 nm was prepared by depositing a Ti^2+^ precursor onto the FTO substrate and spin-casted at 2500 rpm for 15 s, then the substrate was annealed at 500 °C for 1 h to eliminate any organic solvent and form a compact TiO_2_ thin film. Several drops of the CdS NC solution with different concentrations (5 mg/mL, 10 mg/mL, 15 mg/mL, and 20 mg/mL) were then deposited onto the FTO/TiO_2_ and spin-casted at 3000 rpm for 20 s. Following this, the substrate was transferred to a hot plate and annealed at 150 °C for 10 minutes, then transferred to another hot plate and annealed at 380 °C for 30 min. One wash with isopropanol was used to remove any impurities. The CdTe NCs were then deposited layer by layer onto the FTO/TiO_2_/CdS substrate with a process described previously in [23]. Finally, several drops of saturated CdCl_2_/methanol were put onto the FTO/TiO_2_/CdS/CdTe substrate and spin-casted at 1100 rpm for 20 s, then transferred onto a hot plate at 330–420 °C for 15 min. Sixty nanometers of Au was deposited via thermal evaporation through a shadow mask with an active area of 0.16 cm^2^ to make the electrode contact.

The PCE of the NC solar cells were investigated under an illumination of 100 mW cm^−2^ with an air mass 1.5 (AM 1.5) solar simulator (Oriel model 91192), while the *J*-*V* characteristics were measured with a Keithley 2400. The *C*-*V* (Capacity-Voltage) measurements were taken with an Autolab PGSTAT-30 equipped with an impedance analyzer module. The external quantum efficiency (EQE) of the NC solar cell was measured using Solar Cell Scan 100 (Zolix, Beijing, China). Atomic force microscopy (AFM) images were obtained using a NanoScope NS3A system (Veeko, CA, USA). Transient photovoltage measurements (TPV) were taken by using the OmniFluo system (Zolix, Beijing, China).

## 3. Results and Discussion

**Figure 2:**
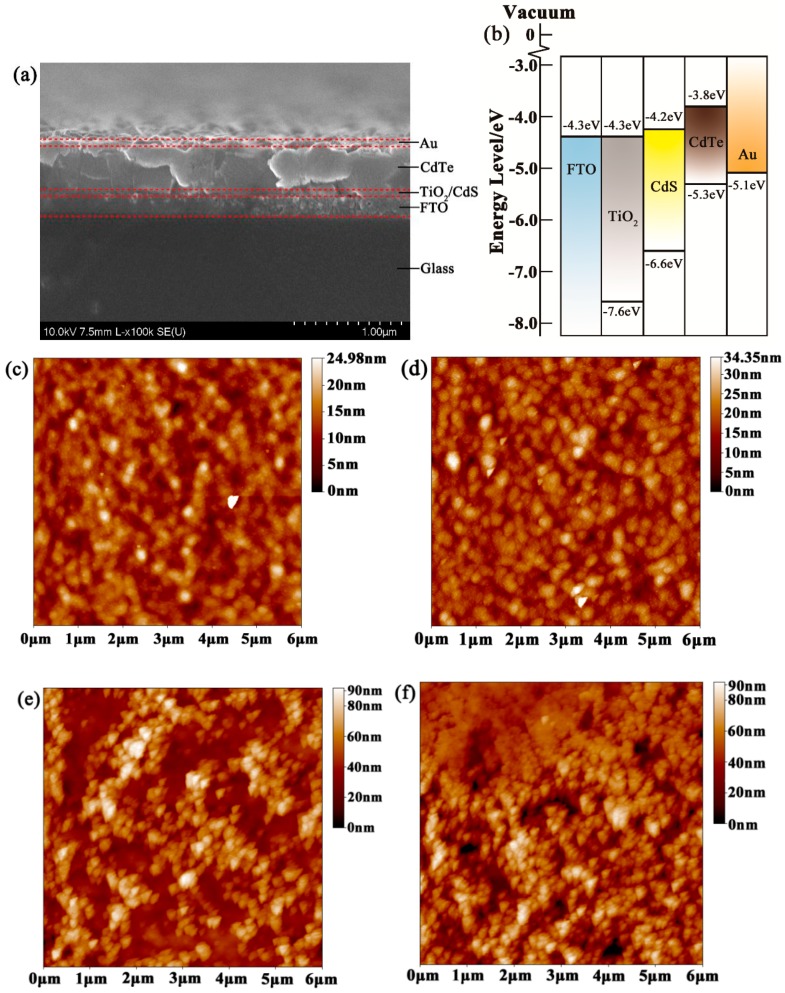
(**a**) Cross-section SEM image of the NC solar cells; (**b**) Energy levels of FTO, TiO_2_, CdS, CdTe, and Au; (**c**) AFM images of FTO/TiO_2_ without CdS; AFM images of FTO/TiO_2_ with CdS; (**d**) 0.78 nm CdS NC; (**e**) 3.74 nm CdS NC; and (**f**) 9.51 nm CdS NC.

The cross-section scanning electron microscope (SEM) image of the optimal CdTe/TiO_2_ NC heterojunction solar cells is shown in Figure 2a. A high-temperature prepared TiO_2_ thin film that was compatible with the FTO substrate was selected as a buffer layer for electron collecting. Zinc-blende CdS NC has a similar structure and size as CdTe NC and was deposited on the TiO_2_ film. A gold electrode was deposited onto the CdTe NC film to collect photo-generated holes. The introduction of the CdS NC film was anticipated to decrease the lattice mismatch and interface defects between the CdTe and TiO_2_. From the energy dispersive spectrum (EDS, Figure S3, supporting information), the emergence of an S element implied that the CdS had been introduced into the NC solar cells. The XRD pattern of the FTO/TiO_2_/CdTe and FTO/TiO_2_/CdS/CdTe thin films is presented in Figure S4; peaks for the zinc blende CdS were found when the CdS NC film was introduced. The band alignment of the FTO, TiO_2_, CdS, CdTe, and Au is presented in Figure 2b. In this device architecture, light passes through the FTO, TiO_2_, then CdS, and is absorbed by the CdTe NC active layer. The photon-generated carriers are separated by the built-in field of CdTe/TiO_2_. Electrons are injected from the conducting band of CdTe to CdS then TiO_2_, and collected by the FTO electrode, while the hole transfers from the valence band of CdTe to the gold electrode. To investigate the morphology changes of the TiO_2_ thin film after the deposition of the CdS thin layer, atomic force microscopy (AFM) was used to characterize the surface images of FTO/TiO_2_/CdS with different thicknesses of CdS NC. As shown in Figure 2c–f, a smooth surface was observed in the case of FTO/TiO_2_/CdS with the thin CdS NC film (0.78 nm, Figure 2d). The TiO_2_ film was totally covered with the CdS NC film when the CdS NC thickness was increased to 3.74 nm (Figure 2e). When the thickness of the CdS NC film reached 9.51 nm, although the TiO_2_ was totally covered by the CdS NC film, the surface was very undulating. It was noted that the root mean squares were 3.01 nm, 4.00 nm, 13.80 nm, and 13.90 nm for a CdS NC thickness increase from 0 to 9.51 nm, respectively. A smooth CdS NC surface is essential to enhance the physical contact between CdS and CdTe and decrease interfacial recombination, leading to improved device performance.

**Figure 3:**
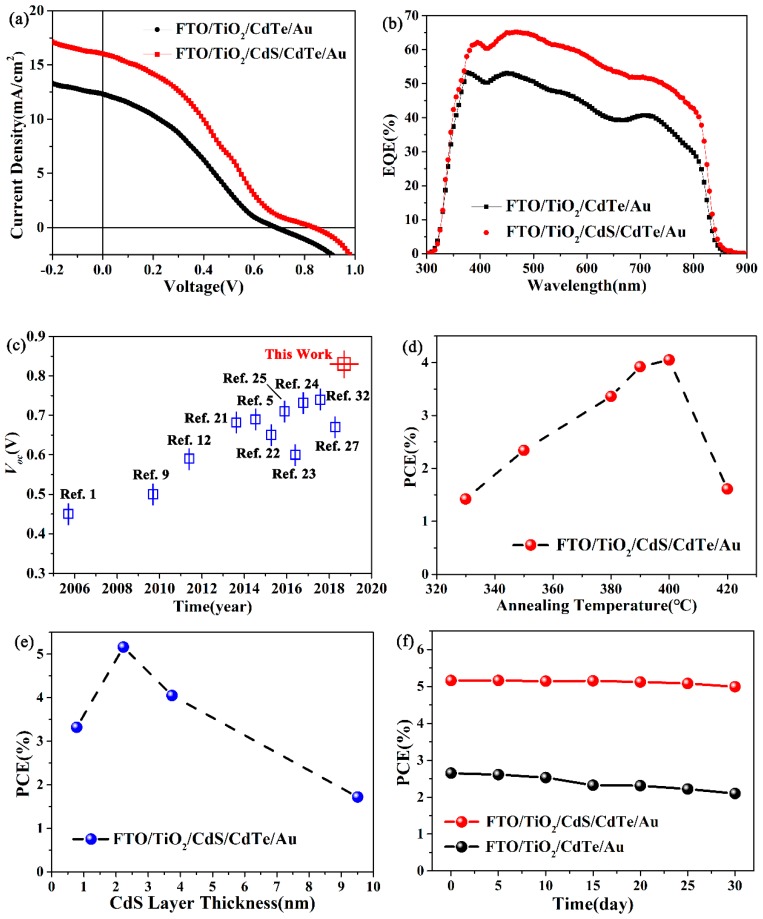
(**a**) *J*-*V* characteristics of the NC solar cells with/without a CdS (3.74 nm) NC interlayer in a structure of FTO/TiO_2_/CdS (with/without)/CdTe/Au. The *J*-*V* curves were measured under 100 mW·cm^−2^ AM 1.5 G illumination, which were corrected by a calibrated Si solar cell. Corresponding (**b**) external quantum efficiency (EQE) spectrum; (**c**) Summary of the *V_oc_* of efficient CdTe NC solar cells reported in the literature; NC solar cells with (**d**) different annealing temperatures and (**e**) different thicknesses of CdS NC film; and (**f**) The stabilized PCEs of NC solar cells with/without a CdS NC interlayer.

To decrease the interface defects between CdTe and TiO_2_, a thin layer of CdS NC was deposited onto the TiO_2_ film with different thicknesses via a solution process. It was reported in our previous works that an optimal annealing temperature for the CdTe NC/TiO_2_ heterojunction was around 400 °C [32]. Figure 3a presents the current density vs. voltage (*J*-*V*) curves of the devices with CdS (3.74 nm) under air mass 1.5 G (AM 1.5 G) illumination, and the detailed parameters are summarized in Table 1. The NC solar cell with a CdS NC interlayer showed a *V_oc_* of 0.83 V, a *J_sc_* of 16.02 mA/cm^2^, and a fill factor (FF) of 30.46%, resulting in a PCE of 4.05%; NC solar cells without the CdS NC interlayer only showed a *V_oc_* of 0.69 V, a *J_sc_* of 12.32 mA/cm^2^, and an FF of 31.17%, leading to a PCE of 2.65%. Therefore, the PCE observed from the NC solar cells with a CdS NC interlayer showed a 52.8% improvement when compared to devices without the CdS NC interlayer. The parallel resistance (*R*_sh_) of the NC solar cells was found to be slightly improved after inserting the CdS NC film, which implies a decreasing carrier recombination for CdS NC devices (Table 1). From the external quantum efficiency (EQE) spectrum (Figure 3b), one can see that the CdS NC interlayer device had a higher photon-to-electron conversion efficiency over the whole wavelength; when they were integrated, current densities of 15.99 mA/cm^2^ and 12.30 mA/cm^2^ were predicted, respectively, which were consistent with our *J*-*V* curves (Figure 3a). It is interesting that the NC devices with a CdS NC interlayer had a drastically improved *V_oc_* (0.83 V for the CdS NC device, 0.69 V for the control device), demonstrating the advantage of the CdS NC interlayer. Figure 3c shows the *V_oc_* of efficient CdTe NC solar cells with the different device structures (the device parameters are summarized in Table 2) that have been reported in recent years. Most devices showed a *V_oc_* below 0.7 V, which was significantly lower than the devices fabricated in this work. This high *V_oc_* value, to the best of our knowledge, is the highest *V_oc_* reported for solution-processed CdTe NC solar cells with different structures. The *V_oc_* obtained in this work was 13–40% higher than that of the conventional CdTe–ZnO NCs solar cells and ~18% higher than that of the inverted CdTe–TiO_2_ NCs solar cells previously reported. The annealing temperature and thickness of the CdS NC film evidently has an influence on the junction quality of the NC solar cells. To investigate the annealing temperature on the performance of the devices, all devices with a 3.74 nm CdS NC interlayer were fabricated at the same conditions except for the final annealing procedure. As shown in Figure 3d (the *J*-*V* curves are presented in Supporting Information Figure S2, while the parameters are summarized in Table 1), the PCE increased linearly with an annealing temperature from 330 °C to 400 °C, then dropped when the annealing temperature was further increased to 420 °C. It was noted that all the devices showed a *V_oc_* up to 0.7 V, and devices annealed at 390 °C/400 °C showed the highest *V_oc_*, surpassing 0.8 V (0.82 V/0.83 V). It is well known that a TiO_2_ thin film prepared by the decomposition of a Ti^2+^ precursor shows a porous structure, which is of benefit for separating the hole/electron pair in the case of dye sensitization solar cells [33]. However, a planar heterojunction is expected for thin film solar cells as a reduced interface area. We anticipated that the incorporation of CdS NC on top of a TiO_2_ thin film would fill the hole of the TiO_2_ film and permit the formation of a smooth and compact CdTe NC film on top of it. Furthermore, when compared to TiO_2_, the CdS NC had a similar size and structure to that of CdTe NC, and therefore a high junction quality was attained in this case due to decreased defects and reduced nonradiative recombination in the interface. On the other hand, due to the low band offset between CdTe and CdS, a high *V_oc_* was expected once the junction quality was improved (improving annealing temperature resulted in a higher junction quality). Further increases of the annealing temperature up to 400 °C may result in the oxidation of CdTe or pin-holes in the CdTe NC thin film, and therefore low device performances will be obtained in this case. It was also found that with increases in annealing temperature from 330 °C to 400 °C, the *R*_s_ decreased from 142.7 Ω·cm^−2^ to ~100 Ω·cm^−2^, while *R*_sh_ decreased from 400 Ω·cm^−2^ to ~150 Ω·cm^−2^. We speculated that with the increase in annealing temperature, the NC may grow larger, therefore resulting in a low *R*_s_. However, as the annealing is conducted under ambient conditions, the surface of CdTe may oxidize, forming CdO at high temperatures, which could increase the series resistance (*R*_s_) of the NC solar cells. On the other hand, aggressive CdCl_2_ treatment at higher temperatures may lead to the formation of some pinholes, which will decrease the *R*_sh_ of the NC solar cells. In CdS thickness experiments, the thickness varied from 0.78 to 9.51 nm, whereas for TiO_2_, the CdTe was fixed at 40 nm and 400 nm with the same structure (FTO/TiO_2_/CdS/CdTe/Au). The PCEs with different CdS NC thicknesses are presented in Figure 3e (the *J*-*V* curves for different CdS NC thicknesses under light are provided in Supporting Information Figure S5, while the detailed photovoltaic parameters are summarized in Table 1). It was evident that the PCEs of the NC solar cells with different thicknesses of CdS were higher than those without a CdS NC interlayer. The PCEs of the NC solar cells increased with a CdS NC from 0 to 2.23 nm, then degraded when the CdS NC thickness exceeded 2.23 nm. The best device was obtained in the case of a 2.23 nm CdS NC interlayer, which showed the following of merits: a *J_sc_* of 17.38 mA/cm^2^, a *V_oc_* of 0.73 V, an FF of 40.67%, and a PCE of 5.16%. The best PCE value was almost one time higher than the control device. It was noted that, although a high PCE was obtained in the 2.23 nm CdS NC device, the *V_oc_* was significantly lower than that of the 3.74 nm CdS NC device. We anticipated that the built-in field was weak for devices with a too-thin CdS thickness due to the inadequate coverage of the TiO_2_ film. The *V_oc_* value was proportional to the built-in field in the NC solar cells. With an increase in the CdS NC thickness, the built-in field of the NC solar cells increased and a high *V_oc_* was expected in this case, which conformed to our experiment results. However, with the increase in the CdS NC film thickness, the *R*_s_ of the NC solar cells increased, which may affect the *J_sc_* and FF of the NC solar cells. Furthermore, the junction quality of CdTe/CdS/TiO_2_ also had significant effects on the FF and the efficiency of the NC solar cells. One of the major issues for solution-processed NC solar cells is device stability. We examined the stability of NC solar cells without a CdS NC interlayer under ambient operating conditions. In this device configuration, the stability of the NC solar cells is mainly related to the CdTe/TiO_2_ interface, the CdTe NC active layer, and the back contact. A device with a CdS NC interlayer maintained 96.7% of its PCE after being placed at ambient conditions (Figure 3f) for 30 days. In contrast, the control device only maintained 79.2% of its initial efficiency. We speculated that the introduction of a CdS NC interlayer restrains the diffusion of defects on the surface of the CdTe NC, therefore improving the stability of the NC solar cells.

**Figure 4:**
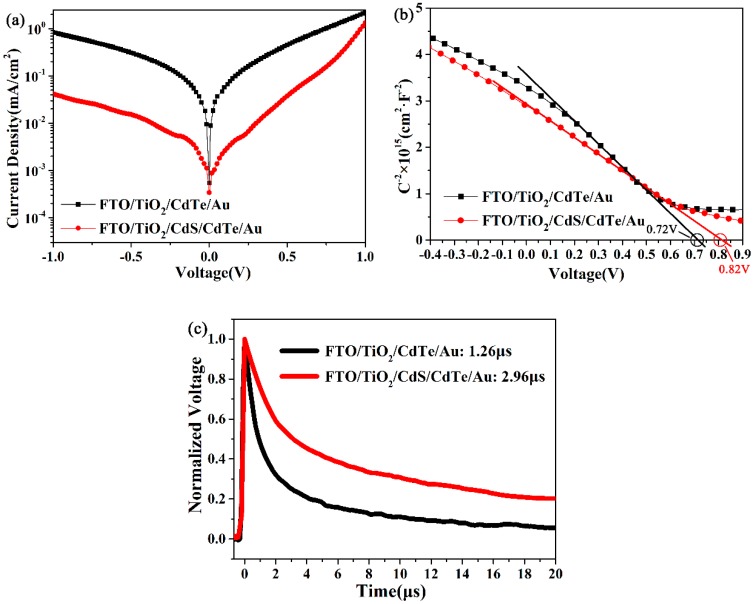
(**a**) *J*-*V* curves of the NC solar cells with/without a CdS thin film under dark; (**b**) Mott–Schottky curves in dark conditions measured at a constant frequency of 1000 Hz for the NC solar cell device with/without a CdS interlayer; and (**c**) Transient photovoltage measurements of the NC solar cells with/without a CdS interlayer.

To gain more insight into the performance improvement in NC solar cells with a CdS NC interlayer, we characterized the *J*-*V* curves under dark. As shown in Figure 4a, the current at the reversed bias from a device with a CdS NC interlayer was almost one order lower than that from a device without a CdS NC interlayer. The low leakage current implied that the CdS NC interlayer could decrease the CdTe/TiO_2_ interface defects and carrier recombination, resulting in a significant improvement in device performance. The built-in potential (*V_bi_*) of the NC solar cells was mainly determined by the *p*-*n* junction between the CdTe and *n*-type partner. Compared to TiO_2_, CdS had a lower lattice mismatch with CdTe and a lower band offset; therefore, a higher *V_oc_* was expected in devices with a CdS NC interlayer, which agreed well to our experimental results. Capacitance–voltage curves (measured at a constant frequency of 1000 Hz) were carried out to investigate the built-in field of NC solar cells with/without a CdS NC interlayer. The *C*^−2^ with voltage (*V*) plotted is shown in Figure 4b. According to the Mott–Schottky equation [34],

(1) $$C^{-2}=\frac{2}{A^{2}{q\varepsilon }_{0}{\varepsilon N}_{A}}\left(V_{bi}-V\right)$$

where *A* is the active area (0.16 cm^2^); *ε* is the relative dielectric constant of CdTe (10.6); *ε_0_* is the vacuum permittivity; and *N_A_* is the net acceptor concentration. The *V_bi_* was extracted at a forward bias from the interception of the fitted line with the *x* axis. A higher *V_bi_* (0.82 V) for devices with a CdS NC interlayer was observed, while this value was 0.72 V for devices without a CdS NC interlayer, which agreed well with their *J*-*V* curves, as shown in Figure 3a. The *N_A_* of NC solar cells calculated from the above formula was ~10^16^/cm^3^. Altering the annealing temperature or using a different structure did not have a significant effect on the *N*_a_ value. To further investigate the effects of the CdS NC interlayer on the recombination process of the NC solar cells, the transient photovoltage (TPV) was used to measure the charge recombination in the NC solar cells with/without a CdS NC interlayer. In the case of TPV measurement, a steady state equilibrium was obtained when the NC solar cells were placed under a white light bias and an additional number of charges were generated by applying another weak laser pulse. As shown in Figure 4c, the charge recombination was characterized by tracking the transient voltage associated with the perturbations in charge population. The charge recombination times for NC devices with/without a CdS NC interlayer were 2.96 μs and 1.26 μs, respectively, which implied a lower charge recombination rate in the CdS NC interlayer device when compared to devices without a CdS NC interlayer. The roll-over of *J-V* at high annealing temperatures (400 °C or above, Figure 3a, Figures S4 and S6), which was also found in our previous work, was also noteworthy [32]. The roll-over mainly originated from the non-ohmic contact between the CdTe and Au, which can be mainly attributed to the large resistance present in the surface of the CdTe NC thin film. We speculated that CdO formed on the surface of the CdTe NC film at high annealing temperatures under ambient conditions. As CdO is an n-type semiconductor material, the device showed a *n*(TiO_2_)–*p*(CdTe)–*n*(CdO) structure, so a *J*-*V* curve with roll-over was very likely to be obtained in this case. Pin-holes that formed in some parts of the NC thin film (due to the large inner stress in NC thin film at high temperature) or the diffusion of CdCl_2_ across the whole NC thin film may also result in device shunt at high annealing temperatures. In this case, a low FF is likely to be obtained. In order to improve the contact quality, we also fabricated an NC device with a MoO_x_/Au back contact or devices that were ozone etched before the Au electrode was deposited. The *J*-*V* curves for the devices with the structure FTO/TiO_2_/CdS/CdTe/MoO_x_ (5 nm)/Au and the devices with different ozone etching times are presented in the Supporting Information Figure S7a,b, while the photovoltaic parameters are summarized in Table S1. Unfortunately, all of these attempts may result in devices shunting, or the degradation of the device performance. Further work should be carried out to eliminate the roll over to improve the performance of the NC solar cells.

## 4. Conclusions

In conclusion, we fabricated efficient CdTe NC/TiO_2_ heterojunction solar cells through a simple layer by layer sintering solution process. The introduction of a thin layer of CdS NC between the CdTe and TiO_2_ resulted in optimized band alignment and reduced the interface defects. Compared to the control devices, drastic improvements in *V_oc_* and PCE were observed for the devices with a CdS NC interlayer. A *V_oc_* as high as 0.83 V was attained by optimizing the thickness of the CdS NC, which was the highest record for solution-processed CdTe NC solar cells. After carefully optimizing the fabrication parameters, we obtained a device with a PCE of 5.16%, showing a 94.7% increase when compared to the control device. Our work here provides a new way to improve the *V_oc_* and performance of CdTe NC solar cells.

## Figures and Tables

**Table 1:** Summary of the photovoltaic parameters of the NC solar cells prepared under different conditions.

Annealing Temperature (°C)	CdS Layer Thickness (nm)	*V_oc_* (V)	*J_sc_* (mA/cm^2^)	FF (%)	PCE (%)	*R_s_* (Ω·cm^−2^)	*R_sh_* (Ω·cm^−2^)
400	0	0.69	12.32	31.17	2.65	96.7	149.1
330	3.74	0.71	6.88	29.07	1.42	142.7	408.0
350	3.74	0.71	12.67	26.01	2.34	96.1	101.4
380	3.74	0.75	14.66	30.56	3.36	101.7	228.4
390	3.74	0.82	15.61	30.62	3.92	103.0	135.8
400	3.74	0.83	16.02	30.46	4.05	108.8	163.4
420	3.74	0.71	9.11	24.89	1.61	148.4	157.8
400	0.78	0.73	14.56	31.24	3.32	93.0	103.9
400	2.23	0.73	17.38	40.67	5.16	51.9	268.3
400	9.51	0.72	11.78	20.28	1.72	126.8	54.4

**Table 2:** Summary of the *V_oc_* obtained in efficient CdTe NC solar cells in the literature.

Device Architecture	*V_oc_* (V)	*J_sc_* (mA/cm^2^)	FF (%)	AM 1.5G Efficiency (%)	Ref.
ITO/CdTe/CdSe/Ca/Al	0.45	13.2	49	2.9	[1]
ITO/CdTe/Al	0.50	4.1	51	1.1	[9]
ITO/CdTe/ZnO/Al	0.59	20.7	56	6.9	[12]
ITO/CdTe/In:ZnO/Al	0.68	25.8	71	12.3	[21]
ITO/CdTe/ZnO/Al	0.69	25.5	64.7	11.3	[5]
ITO/ZnO/CdSe/CdTe/Au	0.65	15.28	58.5	5.81	[22]
ITO/TiO_2_/CdTe/spiro-OMeTAD/Au	0.71	15.82	45.2	5.16	[25]
ITO/ZnO/CdSe/CdSe:CdTe/CdTe/Au	0.60	21.06	49.5	6.25	[23]
ITO/(N_2_H_5_)_2_CdTe_2_/CdTe/ZnO:In/Al	0.73	24.6	71	12.7	[24]
FTO/ZnO/Sb:TiO_2_/CdTe/Au	0.74	11.16	30.13	2.49	[32]
ITO/ZnO/CdS/CdTe/Si-TPA/Au	0.67	20.58	52.76	7.27	[27]
FTO/TiO_2_/CdS/CdTe/Au	0.83	16.02	30.5	4.05	This Work

## Supplementary Materials

The following are available online at http://www.mdpi.com/2079-4991/8/8/614/s1, Figure S1: TEM images of as prepared (a) CdS and (b) CdTe nanocrystal, Figure S2: Transmission spectrum of FTO/TiO_2_/CdS with different thickness of CdS NC film, Figure S3: EDS obtained on the cross-section of CdTe NC solar cells with configuration of FTO/TiO_2_/CdS/CdTe/Au, Figure S4: *J*-*V* characteristic of NC solar cells with different annealing temperatures (all devices with 3.74 nm CdS interlayer), Figure S5: XRD pattern of FTO/TiO_2_ and FTO/TiO_2_/CdS, Figure S6: *J*-*V* characteristic of NC solar cells with different thicknesses of CdS NC film (all devices annealing at 400°C), Figure S7: (a) *J*-*V* curves for NC solar cells with/without MoOx buffer layer (b) *J*-*V* curves for NCs solar cells with different ozone etching times, Table S1: Summarized photovoltaic parameters from Figure S4.
