# Supplementary material for: Efficient CdTe Nanocrystal/TiO2 Hetero-Junction Solar Cells with Open Circuit Voltage Breaking 0.8 V by Incorporating A Thin Layer of CdS Nanocrystal
Source: Nanomaterials (Basel). 2018 Aug 13;8(8):614. doi: 10.3390/nano8080614 (PMC6116231; doi:10.3390/nano8080614)
Supplement: Supplementary file 1 [file nanomaterials-08-00614-s001.pdf]

Supplementary Information

Efficient CdTe Nanocrystal/TiO_2 Hetero-Junction Solar Cells with Open Circuit Voltage Breaking 0.8 V by Incorporating A Thin Layer of CdS Nanocrystal

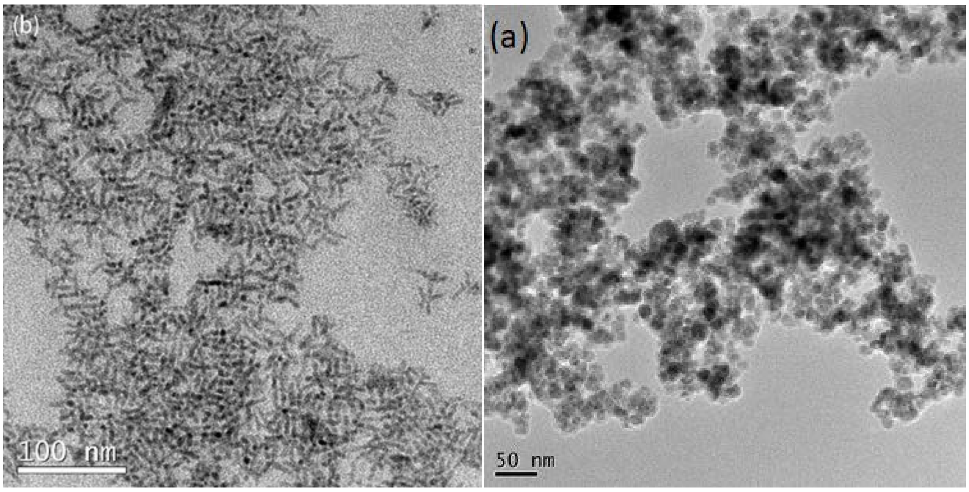

Figure S1 TEM images of as prepared (a) CdS and (b) CdTe nanocrystal.

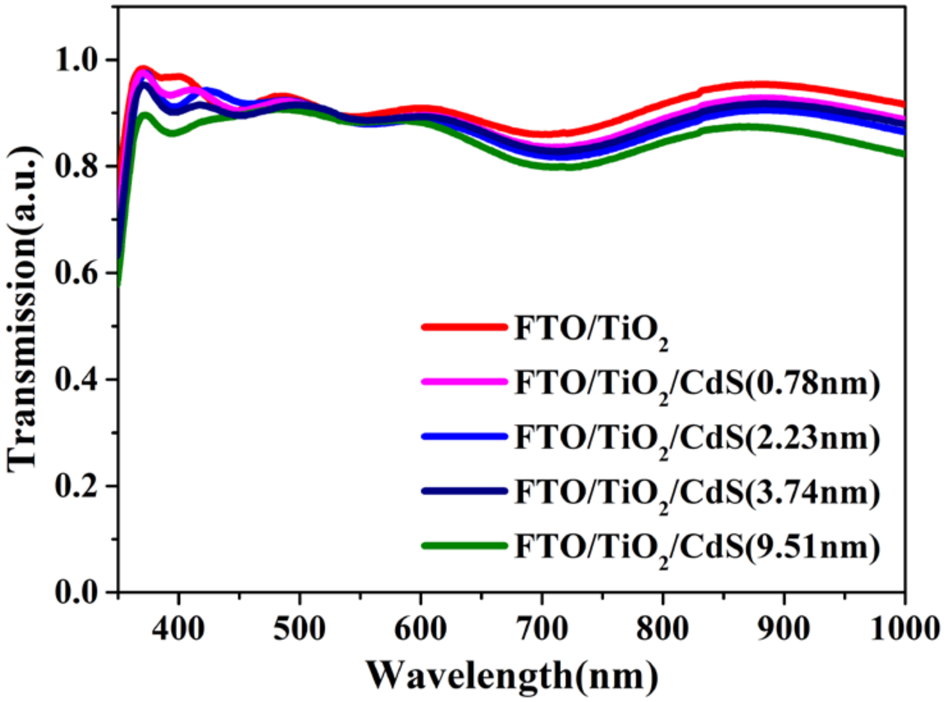

Figure S2 Transmission spectrum of FTO/TiO_2/CdS with different thickness of CdS NC film.

Element	k Value	wt%	wt% Sigma
C	0.00008	4.28	0.46
O	0.00009	5.5	0.42
Na	0.00002	0.6	0.15
Al	0.00004	1.2	0.14
Si	0.00003	0.72	0.13
S	0.00001	0.32	0.26
Cl	0.00003	0.79	0.17
Ti	0.00004	1	0.4
Cd	0.00092	25.29	0.66
Te	0.00068	20.03	0.61
Au	0.00141	40.27	0.95

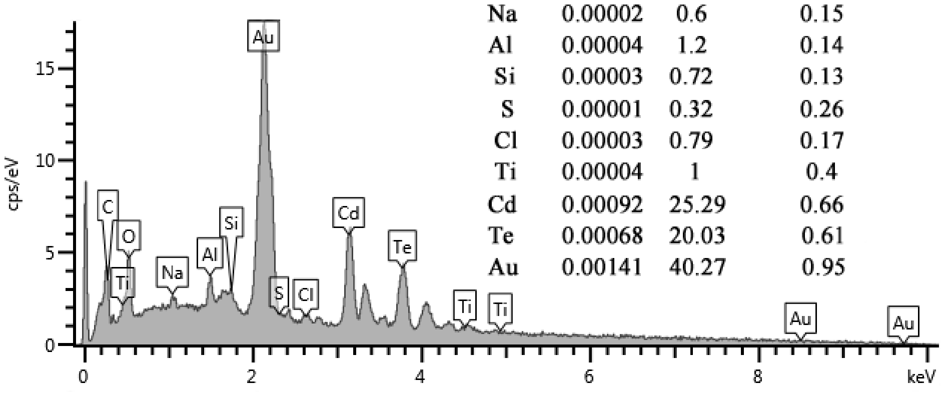

Figure S3 EDS obtained on the cross-section of CdTe NC solar cells with configuration of FTO/TiO_2/CdS/CdTe/Au.

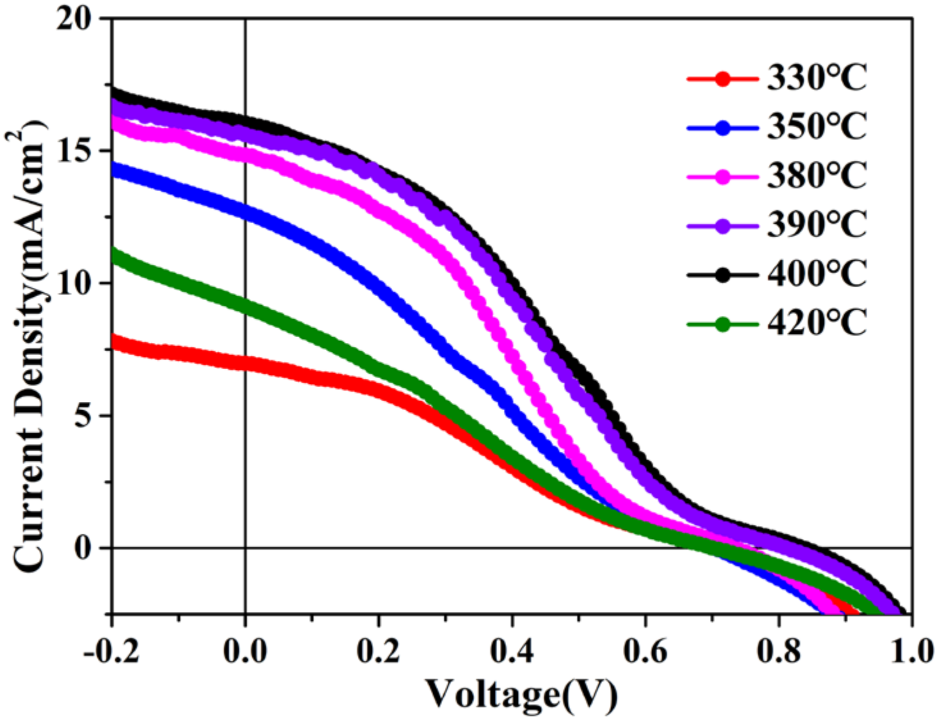

Figure S4 *J-V* characteristic of NC solar cells with different annealing temperatures (all devices with 3.74 nm CdS interlayer).

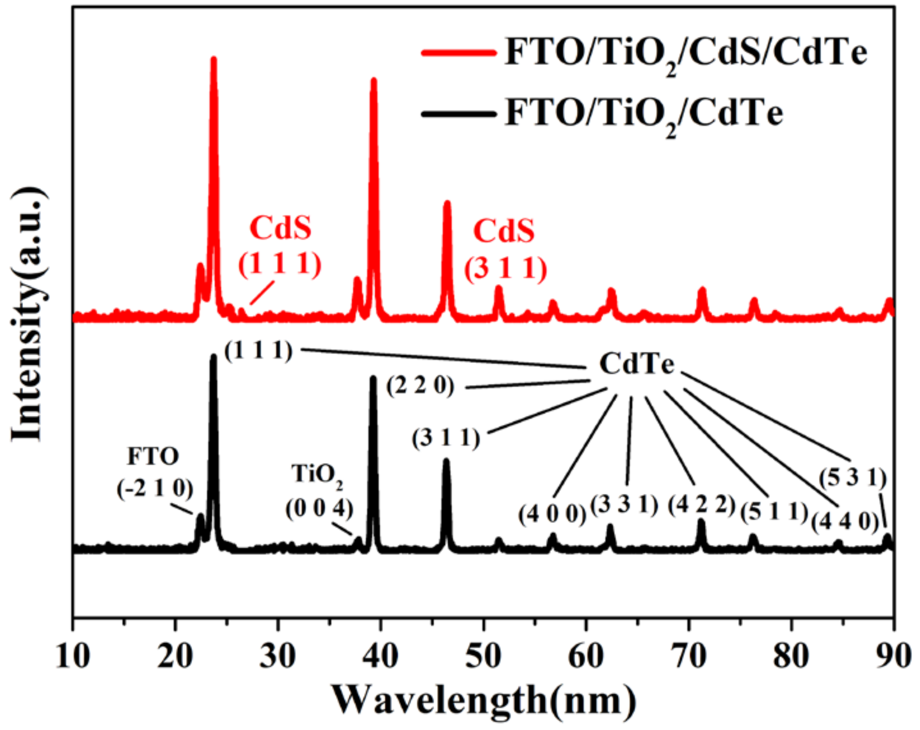

Figure S5 XRD pattern of FTO/TiO_2 and FTO/TiO_2/CdS.

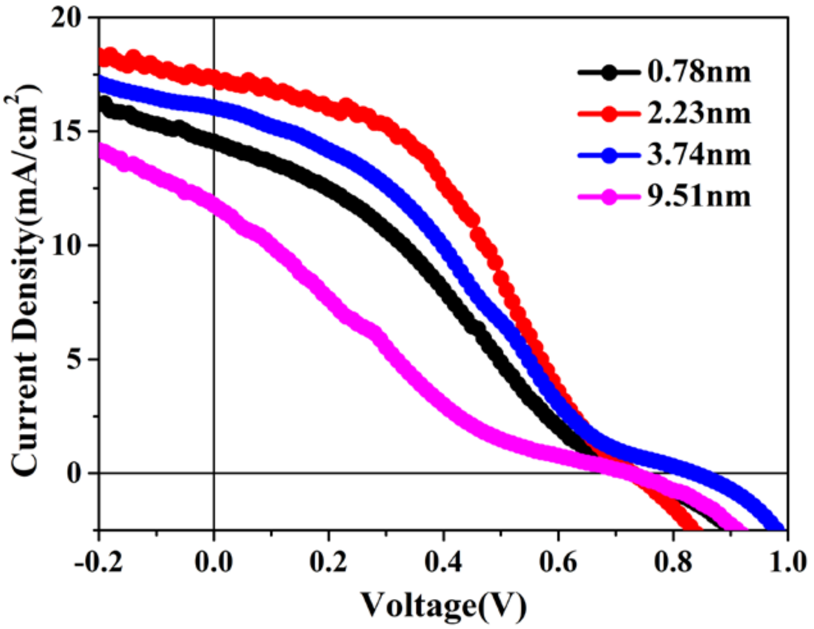

Figure S6 *J-V* characteristic of NC solar cells with different thicknesses of CdS NC film (all devices annealing at 400°C).

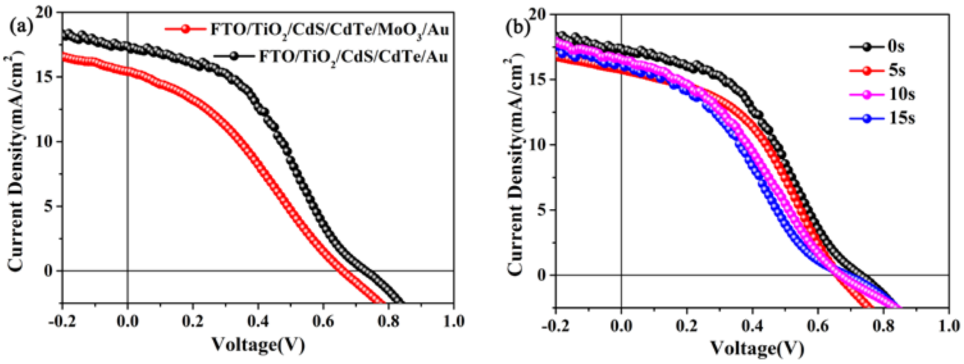

Figure S7 (a) *J-V* curves for NC solar cells with/without MoO_x buffer layer (b) *J-V* curves for NCs solar cells with different ozone etching times.

Table S1 Summarized photovoltaic parameters from Figure S4.

Device Architecture	Etching Time (s)	V_{oc} (V)	J_{sc} (mA/cm^2)	FF (%)	PCE (%)	R_s (Ω·cm^2)	R_{sh}(Ω·cm^2)
$FTO/TiO_2/CdS/CdTe/Au$	0	0.73	17.37	40.69	5.16	51.9	268.3
$FTO/TiO_2/CdS/CdTe/MoO_3/Au$	0	0.66	15.41	33.92	3.45	42.9	407.5
$FTO/TiO_2/CdS/CdTe/Au$	5	0.67	15.79	42.91	4.54	32.3	203.3
$FTO/TiO_2/CdS/CdTe/Au$	10	0.67	16.55	35.89	3.98	51.4	96.6
$FTO/TiO_2/CdS/CdTe/Au$	15	0.69	16.36	32.69	3.69	96.0	157.8
